# Unlocking Superior MFH Performance Below Hergt’s Biological Safety Limit: SPION-Based Magnetic Nanoplatforms Deliver High Heating Efficiency at Low AMF

**DOI:** 10.3390/bioengineering12070715

**Published:** 2025-06-30

**Authors:** Atul Sudame, Dipak Maity

**Affiliations:** 1 Department of Mechanical Engineering, School of Engineering, Shiv Nadar Institution of Eminence, Gautam Buddha Nagar, Greater Noida 201314, India; am465@snu.edu.in; 2 Integrated Nanosystems Development Institute, Indiana University Indianapolis, Indianapolis, IN 46202, USA; 3 Department of Chemistry and Chemical Biology, Indiana University Indianapolis, Indianapolis, IN 46202, USA

**Keywords:** SPIONs, magnetic fluid hyperthermia, PLGA nanoparticles, alternating magnetic field, drug delivery, cancer therapy

## Abstract

Superparamagnetic iron oxide nanoparticles (SPIONs) have gained significant attention for Magnetic Fluid Hyperthermia (MFH)-based cancer therapy. However, achieving high heating efficiency under a biologically safe Alternating Magnetic Field (AMF) remains a challenge. This study investigates the synthesis and optimization of SPIONs encapsulated in TPGS-stabilized PLGA nanoparticles (TPS-NPs) using a modified single emulsion solvent evaporation (M-SESE) method. The aim was to achieve efficient magnetic heating under biologically safe AMF conditions while maintaining biocompatibility and colloidal stability, making these magnetic nanoplatforms suitable for MFH-based cancer treatment. TPS-NPs were characterized using various techniques, including Dynamic Light Scattering (DLS), Atomic Force Microscopy (AFM), Transmission Electron Microscopy (TEM), and Superconducting Quantum Interference Device (SQUID) magnetometry, to evaluate their hydrodynamic size (Dh), zeta potential (ζ), encapsulation efficiency, and superparamagnetic properties. Calorimetric MFH studies demonstrated superior heating efficiency, with Specific Absorption Rate (SAR) and Intrinsic Loss Power (ILP) values optimized at an AMF of 4.1 GAm^−1^s^−1^, remaining within Hergt’s biological safety limit (~5 GAm^−1^s^−1^). These findings suggest that SPION-encapsulated TPS-NPs exhibit enhanced heat induction, making them promising candidates for MFH-based cancer therapy. The study highlights their potential as multifunctional nanoplatforms for magnetic hyperthermia therapy, paving the way for clinical translation in oncology for advanced cancer treatment.

## 1. Introduction

Cancer remains one of the leading causes of mortality worldwide, with chemotherapy being the conventional treatment approach [1]. However, its efficacy is often hindered by severe systemic toxicity [2], drug resistance [3], and poor tumor penetration [4]. These challenges highlight the need for alternative treatment strategies that enhance therapeutic precision while minimizing adverse effects. In recent years, nanotechnology has emerged as a promising avenue for improving cancer treatment by enabling targeted and efficient therapeutic interventions [5,6]. Among various nanocarriers, poly(lactic-co-glycolic acid) (PLGA)-based polymeric nanoparticles have gained significant attention due to their biocompatibility, biodegradability, and FDA approval for biomedical applications. These nanoparticles provide an excellent platform for controlled drug delivery, improved bioavailability, and enhanced stability of therapeutic agents [7,8]

One of the most promising nanotechnology-based approaches in cancer therapy involves the use of superparamagnetic iron oxide nanoparticles (SPIONs) for Magnetic Fluid Hyperthermia (MFH) [9]. Unlike conventional hyperthermia techniques, which rely on external heat sources, MFH leverages the magnetic properties of SPIONs to generate localized heat when exposed to an alternating magnetic field (AMF). This allows for the precise thermal ablation of tumor cells while minimizing damage to surrounding healthy tissues [10,11]. Additionally, SPIONs possess tunable surface chemistry, enabling functionalization with biocompatible coatings that enhance stability, dispersibility, and targeted therapeutic potential [12,13]. These properties make SPIONs a versatile and highly efficient platform for advanced oncological applications, particularly in solid tumor treatment.

Despite the potential of MFH, several challenges remain in optimizing SPION-based nanoplatforms for clinical applications. A major limitation is the encapsulation of SPIONs within polymeric matrices, which can compromise their magnetic properties and reduce heating efficiency under AMF exposure. Additionally, achieving uniform SPION distribution while maintaining high encapsulation efficiency (EE%) is technically challenging, as aggregation can occur, leading to suboptimal therapeutic performance [14,15,16]. Moreover, strong AMF fields and high SPION concentrations, while necessary for effective hyperthermia, may introduce safety concerns, including the unintended heating of the surrounding healthy tissues [17,18,19]. These limitations necessitate the development of advanced nanoplatforms that optimize the balance between biocompatibility, stability, and magnetic performance [20]. To address these challenges, we propose a novel smart superparamagnetic polymeric nanoplatform (SPNP): TPGS-stabilized PLGA nanoparticles (TPS-NPs) encapsulating Oleic-Acid-and-Oleylamine-coated SPIONs (OLM-SPIONs). TPGS (D-α-tocopherol polyethylene glycol 1000 succinate), an FDA-approved surfactant, enhances nanoparticle stability, prevents aggregation, and improves SPION encapsulation efficiency while preserving the magnetic response. Additionally, TPGS provides improved dispersibility in aqueous environments, ensuring a more homogeneous nanoparticle suspension, which is critical for effective MFH applications [21,22,23,24].

In this study, TPS-NPs were synthesized using an our modified single emulsion solvent evaporation (M-SESE) method to achieve controlled particle size, high SPION encapsulation, and enhanced heating efficiency within a biologically safe AMF range [25]. The primary objective of this study was to develop a clinically translatable, biocompatible SPION-based nanoplatform that delivers efficient Magnetic Fluid Hyperthermia (MFH) performance under biologically safe alternating magnetic field (AMF) conditions specifically, within Hergt’s safety threshold (H×f ≤ ~5 GAm^−1^s^−1^) while maintaining stability, uniformity, and encapsulation efficiency. Unlike previous studies focused on maximizing ILP values, our goal was to achieve reliable thermal responses within safe AMF limits using a polymeric nanoplatform suitable for potential future in vivo applications. To validate their potential, we systematically characterized TPS-NPs using multiple techniques, including Dynamic Light Scattering (DLS) for hydrodynamic size and dispersibility, Transmission Electron Microscopy (TEM) and Atomic Force Microscopy (AFM) for morphology, and SQUID magnetometry for magnetic properties. Calorimetric MFH studies were conducted to assess heating performance at an AMF strength of 4.1 GAm^−1^s^−1^, which remains within Hergt’s biological safety limit (~5 GAm^−1^s^−1^) [17]. The results demonstrated that TPS-NPs achieve significantly improved Specific Absorption Rate (SAR) and Intrinsic Loss Power (ILP) values compared to conventional SPION-based platforms. These findings highlight the potential of TPS-NPs as a promising and safe nanoplatform for MFH-mediated cancer therapy, particularly in clinical contexts where balancing heating efficiency with biological safety and multifunctionality is essential.

## 2. Materials and Methods

### 2.1. Materials

High-purity chemicals were used to ensure the reproducibility and reliability of nanoparticle synthesis. Iron (III) acetylacetonate (Fe(acac)_3_), oleic acid (OA), and oleylamine (OM) were purchased from Sigma Aldrich (St. Louis, MI, USA) and utilized as precursors for OLM-SPION synthesis. Poly(lactic-co-glycolic acid) (PLGA) (Resomer RG 502H—acid-terminated, lactide/glycolide 50:50, Mw 7–17 kDa) and D-α-tocopherol polyethylene glycol 1000 succinate (TPGS, MW = 1513 Da) were also procured from Sigma Aldrich (St. Louis, MI, USA) for TPS-NP formulation. Organic solvents, including chloroform and tetrahydrofuran (THF), were purchased from Fisher Scientific (Hampton, NH, USA). All reagents were selected based on their suitability for nanoparticle synthesis, encapsulation, and characterization processes.

### 2.2. Synthesis of TPGS-Stabilized PLGA Nanoparticles (TPS-NPs)

**Figure 1:**
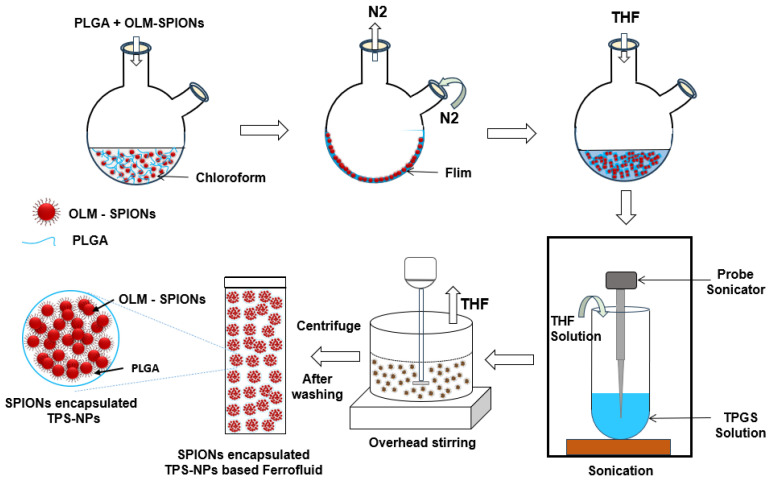
Schematic representation of the preparation of TPGS-stabilized PLGA nanoparticles (TPS-NPs) by the modified single emulsion solvent evaporation (M-SESE) method. The method allows for the synthesis of both blank TPS-NPs (without SPIONs) and SPION-loaded TPS-NPs, depending on whether OLM-SPIONs are included in the initial organic phase.

The TPS-NPs encapsulating the OLM-SPIONs were prepared using our previously reported modified single emulsion solvent evaporation (M-SESE) method (illustrated in Figure 1), which was optimized to achieve controlled nanoparticle size and high encapsulation efficiency [25]. Initially, PLGA (10 mg) was dissolved in 10 mL of chloroform in a two-neck round-bottom flask. To form a uniform polymeric film, the solvent was removed by nitrogen gas bubbling, followed by overnight drying in a vacuum desiccator to eliminate residual solvents completely. The dried polymeric film was then re-dissolved in tetrahydrofuran (THF, 2 mL) and mixed with an aqueous TPGS solution (0.5–1 wt%) under ultrasonication (250W, Branson-Digital Sonifier SFX250 (Emerson Electric Co., St. Louis, MO, USA)) for 5 min to create an emulsion.

The emulsified solution was stirred for 4 hours under fume-hood conditions to facilitate THF evaporation, leading to the formation of a stable colloidal suspension of empty TPS-NPs. The nanoparticles were collected via centrifugation, washed thoroughly with Milli-Q water to remove any unreacted materials, and re-suspended for further characterization. A portion of the suspension was freeze-dried using a freeze dryer (Sub-Zero Instruments, Chennai, India) to obtain powdered TPS-NPs. This process resulted in empty TPS-NP samples (designated S1–S6) with varying TPGS concentrations, as detailed in Table 1. The optimal formulation for empty TPS-NPs (S4 and S5) was selected based on key parameters such as smaller particle size and lower polydispersity index (PDI) to ensure favorable characteristics for subsequent SPION loading.

Building upon the optimized empty TPS-NPs, SPION-encapsulated TPS-NPs (S7–S14) were synthesized following the same M-SESE method, with the inclusion of OLM-SPIONs in varying concentrations (2–8 mg_Fe_) during the polymer film formation step (refer to Table 1). The selection of the optimal formulation was based on achieving smaller hydrodynamic size (Dh), lower PDI, and higher SPION encapsulation efficiency (EE%). The synthesized nanoparticles underwent thorough washing to remove excess OLM-SPIONs and were subsequently freeze-dried for storage and further characterization. This optimized synthesis process ensured the fabrication of a broad range of TPS-NP formulations with controlled SPION loading, making them highly suitable for MFH applications.

### 2.3. Characterization

The structural and magnetic properties of the OLM-SPIONs were analyzed before their encapsulation within the TPS-NPs to ensure their suitability for Magnetic Fluid Hyperthermia (MFH) applications. Transmission Electron Microscopy (TEM-JEM-2100, JEOL Ltd., Tokyo, Japan) was used to determine particle size, while Superconducting Quantum Interference Device (SQUID–MPMS 3, Quantum Design, San Diego, CA, USA) magnetometry assessed their superparamagnetic properties. Additionally, Thermogravimetric Analysis (TGA; Mettler Toledo, TGA 2 STARe System, Columbus, OH, USA), was performed to evaluate the organic coating layer on the OLM-SPIONs, ensuring stability during encapsulation. Following SPION encapsulation within the TPS-NPs (samples S7–S14), Dynamic Light Scattering (DLS, nanoPartica SZ100-Z, Horiba Ltd., Kyoto, Japan) was employed to measure the hydrodynamic size (Dh), polydispersity index (PDI), and zeta potential (ζ), providing insights into nanoparticle size distribution, colloidal stability, and surface charge. The encapsulation efficiency (EE%) of the OLM-SPIONs within the TPS-NPs was quantified using a potassium thiocyanate-based (KTP) assay to quantify the iron content (Fe) [26]. In this method, nanoparticles were dissolved in hydrochloric acid (HCl), and the released iron was complexed with potassium thiocyanate. The absorbance of the resulting iron–thiocyanate complex was measured at 474 nm using UV–Vis spectroscopy (Cary 8454, Agilent Technologies, Santa Clara, CA, USA). The EE% was calculated using the following equation:

(1) $$\mathrm{EE}\;\%=\frac{\mathrm{Amount}\;\mathrm{of}\;\mathrm{Fe}\;\mathrm{encapsulated}\;\mathrm{inside}\;\mathrm{TPS}-\mathrm{NPs}}{\mathrm{Amount}\;\mathrm{of}\;\mathrm{Fe}\;\mathrm{initially}\;\mathrm{added}\;}$$

Further structural and surface analyses were conducted to evaluate the morphology and composition of the TPS-NPs. Atomic Force Microscopy (AFM Oxford Instrument, MFP3D-Origin, Abingdon, United Kingdom) was utilized to assess the surface topography, smoothness, and size distribution of the nanoparticles. High-resolution TEM was performed to confirm the encapsulation of the OLM-SPIONs within the polymeric matrix. Magnetic properties, crucial for MFH applications, were further analyzed using SQUID magnetometry to determine saturation magnetization (Ms) and to confirm the retention of the OLM-SPIONs’ superparamagnetic behavior post-encapsulation. SPION loading within the TPS-NPs was quantified via TGA, where weight loss as a function of temperature was used to estimate SPION content. Differential Scanning Calorimetry (DSC; Mettler Toledo DSC 3 STARe System, Columbus, OH, USA) was conducted to investigate the physical state of the OLM-SPIONs within the polymer matrix, providing insights into any potential interactions that may influence their thermal stability and performance in MFH applications.

### 2.4. Calorimetric MFH

The heating performance of the OLM-SPION-encapsulated TPS-NPs was evaluated using calorimetric Magnetic Fluid Hyperthermia (MFH) studies conducted on a MagneTherm™ (nanoTherics, Warrington, United Kingdom) hyperthermia instrument. The experimental setup was adapted from prior reports, with modifications to suit the optimization of SPION concentration and the applied AMF parameters specific to this study [25,27,28]. In each experiment, a 1 mL aqueous suspension of TPS-NPs containing a known concentration of SPIONs (quantified in terms of iron content) was transferred into a sample vial. Temperature changes were continuously monitored over a 60-min period using an single-channel fiber optic temperature probe (OSENSA Innovations Corp., Coquitlam, BC, Canada) integrated with the MagneTherm™ instrument. The probe was inserted into the sample vial, which was placed at the center of the AMF induction coil. An alternating magnetic field was applied with an amplitude of H = 8.6 kA/m and a frequency of f = 475.5 kHz, resulting in an H×f product of approximately 4.1 GAm^−1^s^−1^—well within Hergt’s biological safety threshold (~5 GAm^−1^s^−1^). This ensured that the experiments were conducted under clinically acceptable AMF conditions. To assess MFH efficiency, two quantitative parameters were calculated: Specific Absorption Rate (SAR) and Intrinsic Loss Power (ILP). The SAR (in W/g_Fe_) was determined from the initial slope of the temperature–time curve during the early heating phase. It was calculated using the following equation:

(2) $$\mathrm{SAR}=\frac{C_{\mathrm{samp}}\times \rho_{\mathrm{samp}}}{m_{\mathrm{Fe}}}\;\frac{∆T}{∆t}$$

where C_samp_ is the specific heat capacity of water, ρ_samp_ is the density of water (in which the SPIONs are dispersed), m_Fe_ is the mass of iron in the sample, and ΔT/Δt is the initial temperature rise over time. This method allows for a direct estimation of the energy converted to heat per unit mass of iron in the SPION-encapsulated TP-NP suspension. To facilitate comparisons across different AMF systems and studies, ILP (in nHm^2^/kg) was calculated by normalizing the SAR to the square of the field amplitude and the frequency of the AMF using the following relation:

(3) $$\mathrm{ILP}=\frac{\mathrm{SAR}}{H^{2}f}$$

where H is expressed in kA/m and f in Hz. ILP serves as a field-independent metric, enabling a standardized evaluation of the heating efficiency of magnetic nanomaterials under varying AMF conditions [29,30].

## 3. Results

### 3.1. OLM-SPIONs

Hydrophobic OLM-SPIONs with a monodisperse size distribution were freshly synthesized using our previously reported methodology, yielding nanoparticles with an average diameter of approximately 10 nm. Magnetic characterization confirmed their superparamagnetic behavior, with saturation magnetization (Ms) values of 66.6 emu/g at 10 K and 59.1 emu/g at 300 K, as documented in our earlier publication [25].

### 3.2. TPGS-Stabilized PLGA Nanoparticles (TPS-NPs)

#### Optimization of Empty and SPION-Encapsulated TPS-NPs

- Effects of concentration of stabilizer

The influence of TPGS concentration on the hydrodynamic diameter (Dh) and polydispersity index (PDI) of the TPS-NPs was systematically evaluated while keeping the PLGA content fixed at 10 mg. The concentration of the stabilizer (TPGS) was varied from 0 to 2 wt%, resulting in the synthesis of TPS-NP samples labeled S1, S2, S4, S5, and S6. The measured Dh values were 176 ± 65 nm, 168 ± 24 nm, 156 ± 7 nm, 151 ± 5 nm, 166 ± 8 nm, and 175 ± 51 nm, respectively, with corresponding PDI values of 0.24 ± 0.12, 0.22 ± 0.15, 0.18 ± 0.02, 0.14 ± 0.05, 0.11 ± 0.04, and 0.20 ± 0.11 (refer to Table 1 and Figure S1i for details). Among these, samples S4 (0.5 wt% TPGS) and S5 (1 wt% TPGS) were found to exhibit the most favorable characteristics. While S4 showed the smallest Dh (151 ± 5 nm) and a low PDI (0.14 ± 0.05), S5 exhibited the lowest PDI (0.11 ± 0.04) with a slightly higher Dh (166 ± 8 nm). This combination of relatively small particle size and narrow size distribution suggests superior colloidal uniformity and formulation stability. Therefore, both S4 and S5 were selected for the subsequent encapsulation of OLM-SPIONs.

- Effects of OLM-SPION concentration

To evaluate the impact of SPION content on nanoparticle properties, the OLM-SPIONs were encapsulated in the previously optimized TPS-NP formulations (S4 and S5) using increasing SPION feed amounts (2 to 8 mg_Fe_), while keeping PLGA (10 mg) and TPGS (0.5 or 1 wt%) constant. The Dh and PDI values for 0.5 wt% TPGS samples (S7–S10) ranged from 106 ± 3 to 151 ± 5 nm and 0.13 ± 0.02 to 0.32 ± 0.02, respectively. For the 1 wt% TPGS group (S11–S14), Dh ranged from 113 ± 4 to 171 ± 7 nm, with PDI values from 0.15 ± 0.04 to 0.35 ± 0.11 (Table 1; Supplementary Figure S1(ii–iii). Encapsulation efficiency (EE%) remained relatively stable across all SPION concentrations, with values ranging from 62.5% to ~70.8%. However, because 2 and 4 mg_Fe_ formulations involved lower initial SPION amounts, the total SPION content in the resulting nanoparticles was lower despite good EE%. In contrast, samples S9 (6 mg_Fe_, 0.5 wt% TPGS) and S13 (6 mg_Fe_, 1 wt% TPGS) achieved optimal overall loading while maintaining compact size (~144–149 nm), low PDI (~0.13–0.15), and high EE%, and were thus selected for further MFH evaluation.

**Table 1:** Impacts of various factors on the hydrodynamic sizes (Dh) and encapsulation efficiencies (EE%) of empty and SPION-encapsulated TPS-NPs.

Sr. No.	PLGA (mg)	TPGS % (*w*/*v*)	OLM-SPIONs (mg_Fe_)	Mean Hydrodynamic Size (nm)	PDI	Fe (EE %)	Zeta Potential (ζ) (mv)
Empty TPS-NPs							
S1	10	0	NA	176 ± 65	0.24 ± 0.12	NA	-
S2	10	0.05	NA	168 ± 24	0.22 ± 0.15	NA	-
S3	10	0.1	NA	156 ± 7	0.18 ± 0.02	NA	−
S4	10	0.5	NA	151 ± 5	0.14 ± 0.05	NA	−56.4
S5	10	1	NA	166 ± 8	0.11± 0.04	NA	−60.1
S6	10	2	NA	175 ± 51	0.20 ± 0.11	NA	−
SPION-encapsulated TPS-NPs (via 0.5 Wt % TPGS)							
S7	10	0.5	2	106 ± 3	0.32 ± 0.02	~66.7	−23.8
S8	10	0.5	4	125 ± 2	0.20 ± 0.03	~68.9	−28.0
S9	10	0.5	6	144 ± 3	0.13 ± 0.02	~65.4	−9.7
S10	10	0.5	8	151 ± 5	0.32 ± 0.08	~62.5	−2.2
SPION-encapsulated TPS-NPs (via 1 Wt % TPGS)							
S11	10	1	2	113 ± 4	0.21 ± 0.03	~70.8	−34.4
S12	10	1	4	127 ± 6	0.20 ± 0.04	~69.6	−29.2
S13	10	1	6	149 ± 2	0.15 ± 0.04	~67.1	−10.6
S14	10	1	8	171 ± 7	0.35 ± 0.11	~64.8	−3.7

### 3.3. DLS

#### 3.3.1. Hydrodynamic Size

**Figure 2:**
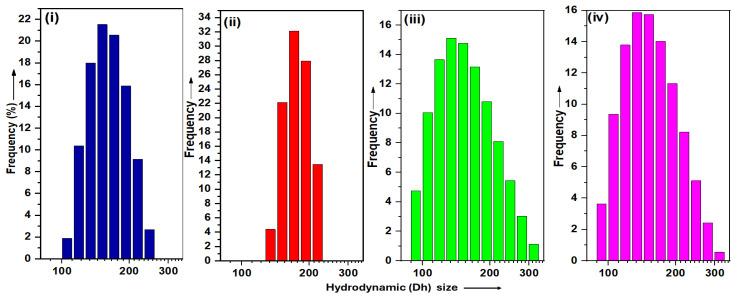
Normalized histograms showing the hydrodynamic size distribution profiles of (**i**) empty TPS-NPs (S4) and (**iii**) SPION-encapsulated TPS-NPs (S9), synthesized using 0.5% *w*/*v* TPGS; and (**ii**) empty TPS-NPs (S5) and (**iv**) SPION-encapsulated TPS-NPs (S13), synthesized using 1% *w*/*v* TPGS. The y-axis represents frequency (%) normalized to a total of 100% for each sample.

Dynamic Light Scattering (DLS) was performed to determine the hydrodynamic size (Dh) and polydispersity index (PDI) of all the TPS-NP samples (S1–S14). The measured values are summarized in Table 1 and illustrated in Figure 2. For the empty TPS-NPs (samples S1–S6), the Dh values ranged from 151 ± 5 nm to 176 ± 65 nm, with corresponding PDI values between 0.14 ± 0.05 and 0.24 ± 0.12. The hydrodynamic size of the SPION-encapsulated TPS-NPs varied based on the TPGS concentration used during synthesis. Samples synthesized with 0.5 wt% TPGS (S7–S10) exhibited Dh values between 106 ± 3 nm and 151 ± 5 nm, with PDI values between 0.13 ± 0.02 to 0.32 ± 0.02. Similarly, samples prepared with 1 wt% TPGS (S11–S14) displayed Dh values ranging from 113 ± 4 nm to 171 ± 7, with a PDI between 0.15 ± 0.04 and 0.35 ± 0.11.

#### 3.3.2. Water Dispersibility

**Figure 3:**
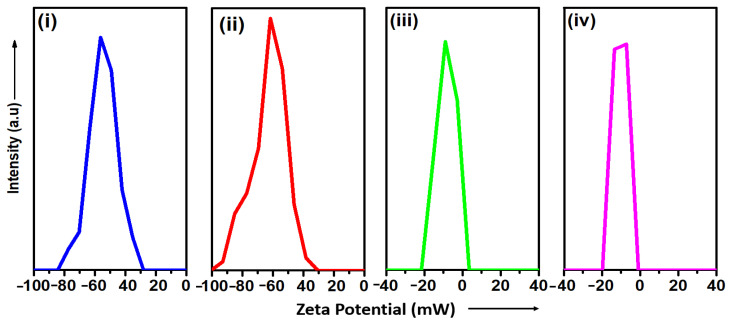
Zeta (ζ) potential profiles for the following samples: (**i**) empty TPS-NPs (S4), (**iii**) SPION-encapsulated TPS-NPs (S9), synthesized via 0.5% Wt TPGS concentration. Similarly, (**ii**) empty TPS-NPs (S5), and (**iv**) SPION-encapsulated TPS-NPs (S13) synthesized via 1% Wt TPGS concentration.

The water dispersibility and surface charge of the TPS-NPs were evaluated using zeta potential (ζ) measurements, with the results summarized in Table 1 and Figure 3. For the empty TPS-NPs (samples S4 and S5), the recorded zeta potential (ζ) values were −56.4 mV and −60.1 mV, respectively. The zeta potential (ζ) of SPION-loaded TPS-NPs varied depending on the TPGS concentration and the amount of OLM-SPIONs incorporated. Samples synthesized with 0.5 wt% TPGS (S7–S10) exhibited zeta potential (ζ) values ranging from −23.8 mV to −2.2 mV, while those prepared with 1 wt% TPGS (S11–S14) displayed values between −34.4 mV and −3.7 mV.

### 3.4. Morphology—AFM and TEM

**Figure 4:**
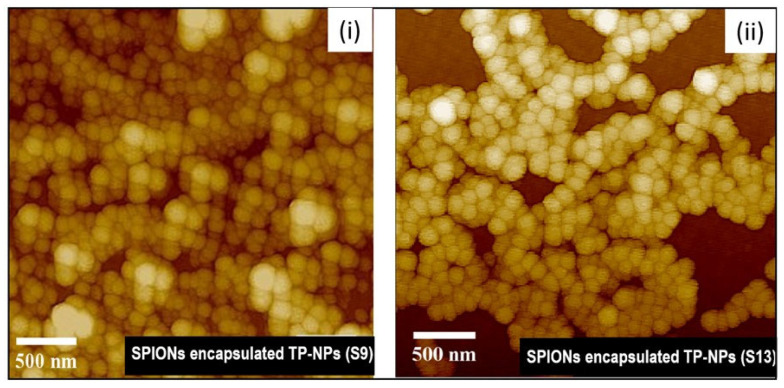

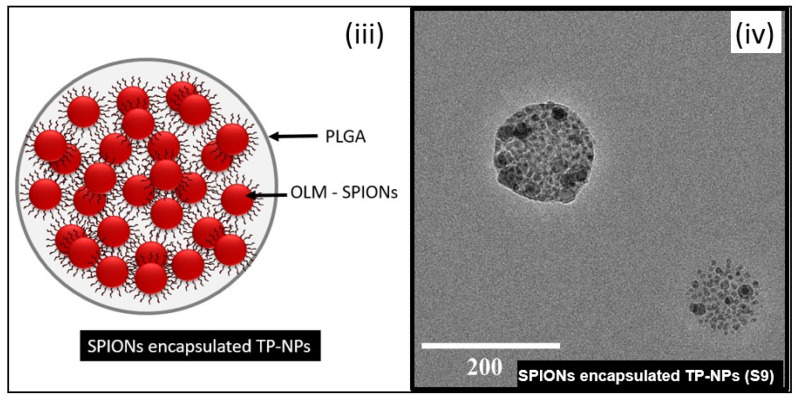
AFM topography images in (**i**,**ii**), depicting SPION-encapsulated TPS-NPs labeled as S9 and S13, respectively. Additionally, (**iii**,**iv**) provide schematic representations of SPION-encapsulated TPS-NPs and TEM images specifically for SPION-encapsulated TPS-NPs (S9), respectively.

The Atomic Force Microscopy (AFM) images of samples S9 and S13 (Figure 4i,ii) revealed that the TPS-NPs exhibited a uniform spherical morphology with a narrow size distribution. The average particle sizes observed via AFM were approximately 140 ± 27.71 nm and 147 ± 30.15 nm for S9 and S13, respectively, indicating consistent nanoparticle formation. Transmission Electron Microscopy (TEM) analysis (Figure 4iii) further confirmed the structural integrity of the nanoparticles and provided visual evidence of successful SPION encapsulation within the TPS-NPs. These morphological characteristics are consistent with the hydrodynamic size (Dh) values obtained from DLS measurements (Table 1).

### 3.5. Magnetic Properties—SQUID

**Figure 5:**
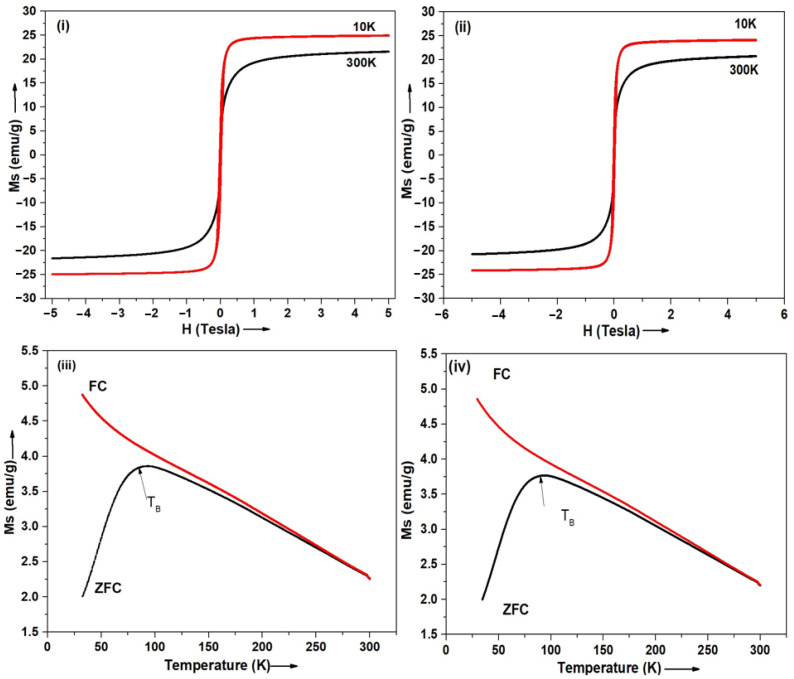
(**i**,**ii**) illustrate the magnetization (M-H) curves for SPION-encapsulated TPS-NPs (S9) and (S13) at 300 K and 10 K. Additionally, figures (**iii**,**iv**) depict the FC and ZFC magnetization curves for SPION-encapsulated TPS-NPs (S9) and (S13) under an applied magnetic field (H) of 100 Oe (where T_B_ is the blocking temperature).

The superparamagnetic properties of the SPION-encapsulated TPS-NPs (S9 and S13) were assessed using a Superconducting Quantum Interference Device (SQUID) magnetometer. The M-H hysteresis loops were recorded at 10 K and 300 K within a field range of −5 T to 5 T (Figure 5i,ii). The absence of coercivity and remanence in the M-H curves confirmed the superparamagnetic behavior of both samples. The saturation magnetization (Ms) values for S9 and S13 were 25.09 ± 0.07 emu/g and 24.13 ± 0.06 emu/g at 10 K, and 21.66 ± 0.03 emu/g and 20.64 ± 0.02 emu/g at 300 K, respectively (Table 2). Field-Cooled (FC) and Zero-Field-Cooled (ZFC) magnetization curves for the SPION-encapsulated TPS-NPs (S9 and S13) at an applied magnetic field of 100 Oe are shown in Figure 5iii,iv. The blocking temperatures (TB) for S9 and S13 were 86.16 K and 90.25 K, respectively, differing from the T_B_ of OLM-SPIONs (~100 K) (Figure S2, Supplementary Information) [25,31,32]. These shifts in T_B_ further confirm the successful encapsulation of the OLM-SPIONs within the TPS-NPs.

### 3.6. Calorimetric MFH

**Figure 6:**
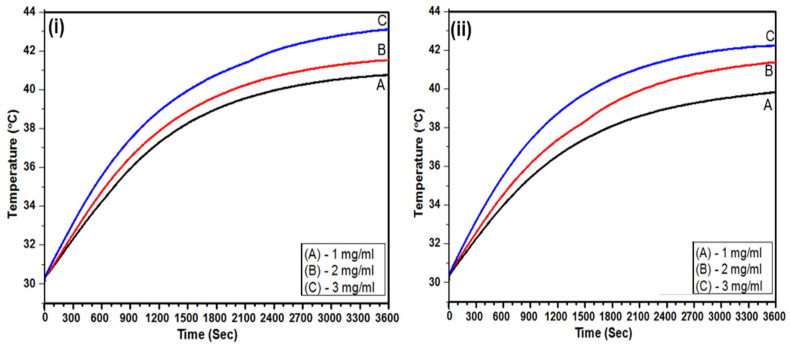
Time-dependent temperature curve of an aqueous suspension (1 mL) for (**i**) SPION-encapsulated TPS-NPs (S9) synthesized using 0.5 Wt % TPGS, and (**ii**) SPION-encapsulated TPS-NPs (S13) synthesized using 1 Wt % TPGS at concentrations of (A) 1, (B) 2, and (C) 3 mg_Fe_/mL when subjected to an AMF with an H×f value of 4.1 GAm^−1^s^−1^ for 60 min.

Calorimetric MFH experiments were conducted to assess the heating efficiency of SPION-encapsulated TPS-NPs (S9 and S13). Aqueous suspensions with iron (Fe) concentrations of 1, 2, and 3 mg_Fe_/mL were exposed to an externally applied AMF. The AMF parameters were set at H = 8.6 kA/m and f = 475.5 kHz, yielding an H×f value of approximately 4.1 GAm^−1^s^−1^, and temperature variations were monitored over 60 min. Figure 6i,ii illustrate the time-dependent temperature curves for S9 and S13. Also, the times required for the samples to reach the therapeutic temperature of 42 °C at various Fe concentrations (1, 2, and 3 mg_Fe_/mL) are summarized in Table 3. The table also includes the respective heating efficiencies in terms of Specific Absorption Rate (SAR) and Intrinsic Loss Power (ILP) values. Notably, for S9 and S13, only the samples at a concentration of 3 mg_Fe_/mL achieved therapeutic temperature (42 °C) within 60 min, reaching this temperature in 39.1 and 49.8 min, respectively, reflecting an approximate 12 °C increase from the initial temperature of 30 °C. Samples at 1 mg_Fe_/mL and 2 mg_Fe_/mL did not reach the therapeutic temperature within the experimental time frame.

The heating performance was further analyzed through SAR and ILP values. The SAR values for S9 and S13 at 3 mg_Fe_/mL were calculated to be 48.8 ± 2.3 W/g_Fe_ and 46.1 *±* 2.1 W/g_Fe_, respectively. The corresponding ILP values were 1.39 ± 0.07 nHm^2^/kg for S9 and 1.31 ± 0.06 nHm^2^/kg for S13 (Table 3). The SAR and ILP values for the 1 mg_Fe_/mL and 2 mg_Fe_/mL samples were not determined, as these samples failed to reach the therapeutic temperature. The results confirm that SPION-encapsulated TPS-NPs (S9 and S13) effectively reached the therapeutic threshold of 42 °C within 39.1 and 49.8 min, respectively, under AMF exposure at H = 8.6 kA/m and f = 475.5 kHz. The H×f value of 4.1 GAm^−1^s^−1^ remained within Hergt’s biological safety limit (~5 GAm^−1^s^−1^), demonstrating a controlled heating response under safe operating conditions for MFH applications.

### 3.7. Thermal Analysis

#### 3.7.1. Thermogravimetric Analysis (TGA)

Thermogravimetric Analysis (TGA) was performed to assess the thermal stability of SPION-encapsulated TPS-NPs (samples S4 and S9). The TGA curves (Figure S3, Supplementary Information) were recorded over a temperature range of 30–600 °C to evaluate the weight loss behavior of the samples. The thermogravimetric profiles exhibited two distinct weight loss phases. The first stage, occurring between 30–200 °C, corresponds to the removal of physically adsorbed water molecules. The second stage, observed between 201–450 °C, is associated with the thermal degradation of the PLGA polymer matrix. The estimated PLGA content in S4 and S9 was determined to be approximately 98.8 wt% and 65.75 wt% (±2 wt%), respectively. The residual weight observed in S9 can be attributed to the presence of encapsulated OLM-SPIONs (Fe_3_O_4_), which aligns with the encapsulation efficiency (EE%) measured using UV–Vis spectroscopy (refer to Table 1).

#### 3.7.2. Differential Scanning Calorimetry (DSC)

DSC analysis was conducted to determine the physical state (amorphous or crystalline) of the encapsulated OLM-SPIONs within the TPS-NPs, as this characteristic can significantly influence their magnetic properties and MFH performance. The DSC curves (Figure S4(i–iii), Supplementary Information) were recorded for raw PLGA polymer, empty TPS-NPs (S4), and SPION-encapsulated TPS-NPs (S9). All samples exhibited distinct endothermic peaks, indicating phase transitions. The PLGA polymer, empty TPS-NPs (S4), and SPION-encapsulated TPS-NPs (S9) displayed peaks at 46.4 °C, 48.6 °C, and 49.29 °C, respectively, suggesting that nanoparticle formation and SPION encapsulation may induce slight alterations in the thermal properties of the polymer matrix. Additionally, the presence of amorphous TPGS in the formulations was confirmed by additional peaks observed at 37.30 °C and 39.12 °C, consistent with previously reported studies [33,34,35].

## 4. Discussion

The development and optimization of TPGS-stabilized PLGA nanoparticles (TPS-NPs) encapsulating SPIONs were systematically conducted to enhance colloidal stability, SPION encapsulation efficiency, and Magnetic Fluid Hyperthermia (MFH) performance. The optimization process began by fine-tuning the stabilizer (TPGS) concentration, a critical factor in controlling particle size, surface charge, and dispersibility in polymeric nanoparticle formulations [36,37]. As demonstrated in DLS analysis, increasing the TPGS concentration from 0 to 1 wt% resulted in a consistent reduction in hydrodynamic diameter (Dh) and polydispersity index (PDI), with an optimal window observed between 0.5 and 1 wt% TPGS (refer Figure S1i). Samples S4 and S5, formulated at 0.5 wt% and 1 wt% TPGS, respectively, demonstrated optimal characteristics in terms of size uniformity and dispersibility—aligning with prior reports that highlight the role of surfactants in reducing interfacial tension and stabilizing polymeric nanoparticles [38,39]. Although S4 exhibited the smallest Dh, S5 achieved the lowest PDI, emphasizing that optimal nanoparticle formulations may result from a balance of multiple parameters rather than a single metric.

Furthermore, the high negative zeta potential values recorded for both samples (−56.4 mV and −60.1 mV) support the notion of electrostatically driven colloidal stabilization. Such surface charge characteristics are crucial for maintaining nanoparticle dispersion in physiological media and minimizing aggregation—factors that are essential for biomedical applications [40]. Hence, S4 and S5 were rationally selected for SPION encapsulation and further Magnetic Fluid Hyperthermia (MFH) evaluation based on a combination of particle size, uniformity, and surface charge stability.

Following the optimization of the empty TPS-NPs, the OLM-SPIONs were encapsulated to assess their impact on the physicochemical characteristics of the nanoplatforms and their potential for Magnetic Fluid Hyperthermia (MFH) applications. As shown in Supplementary Figure S1(ii,iii), all formulations demonstrated consistently high encapsulation efficiencies (EE%), regardless of SPION input concentration. However, at lower inputs (2 and 4 mg_Fe_), the total amount of encapsulated SPIONs was inherently limited due to the smaller initial SPION load, despite good EE%. Conversely, increasing the SPION feed to 8 mg_Fe_ led to undesirable increases in hydrodynamic diameter (Dh) and polydispersity index (PDI), likely due to overloading and agglomeration during encapsulation—an effect commonly reported in SPION–polymer systems [41,42].

Among all formulations, S9 and S13—both containing 6 mg_Fe_ SPIONs and stabilized with 0.5 wt% and 1 wt% TPGS, respectively—emerged as the most promising candidates. These formulations exhibited an optimal balance of key attributes: low Dh (144 ± 3 and 149 ± 2 nm), narrow PDI (0.13 ± 0.02 and 0.15 ± 0.04), and high EE% (~65.4% and ~67.1%). The improved retention of SPIONs at this loading was attributed to efficient polymer–stabilizer interactions that minimized loss during emulsification and solidification [39]. Furthermore, despite SPION incorporation, all TPS-NP samples retained a negative zeta potential (ζ), which is essential for colloidal stability and prolonged dispersion in biological environments [43,44,45]. These findings establish S9 and S13 as promising candidates for MFH applications due to their optimized size, stability, encapsulation efficiency, and surface charge properties.

The morphological analysis of SPION-loaded TPS-NPs using Atomic Force Microscopy (AFM) and Transmission Electron Microscopy (TEM) provided further confirmation of the size distribution and encapsulation of the OLM-SPIONs. AFM images of S9 (~140 ± 27.71 nm) and S13 (~147 ± 30.15 nm) demonstrated a spherical morphology with a uniform size distribution, consistent with the DLS-measured hydrodynamic sizes. These findings align with previously reported studies, where spherical nanoparticles exhibited enhanced circulation time and cellular uptake due to reduced non-specific interactions with biological barriers [46,47]. TEM imaging further confirmed the successful encapsulation of the OLM-SPIONs within the polymeric matrix, as evidenced by dark SPION clusters within the TPS-NPs. This observation aligns with previous reports, reinforcing that the OLM-SPIONs were effectively entrapped within the nanoparticles rather than adsorbed onto their surface [26,48,49,50,51]. These results highlight the effectiveness of the modified single emulsion solvent evaporation (M-SESE) method used in this study, which successfully produced nanoparticles with controlled morphology and high OLM-SPION entrapment—key factors for MFH-based cancer therapy.

The magnetic characterization of SPION-loaded TPS-NPs was conducted using Superconducting Quantum Interference Device (SQUID) magnetometry, confirming their superparamagnetic behavior—an essential property for MFH applications. The M-H hysteresis loops of S9 and S13 at 300 K and 10 K exhibited negligible coercivity and remanence, verifying their superparamagnetic nature, which prevents particle aggregation in the absence of an external magnetic field [52]. The saturation magnetization (Ms) values of S9 (25.09 ± 0.07 emu/g at 10 K, 21.66 ± 0.03 emu/g at 300 K) and S13 (24.13 ± 0.06 emu/g at 10 K, 20.64 ± 0.02 emu/g at 300 K) were lower than those of the original OLM-SPIONs (66.6 emu/g at 10 K, 59.1 emu/g at 300 K). This reduction in Ms is attributed to the non-magnetic PLGA polymer matrix, which dilutes the overall magnetic response—a well-documented phenomenon in polymer-encapsulated SPION formulations [53,54]. However, S9 exhibited slightly higher Ms values than S13, likely due to its smaller hydrodynamic size, which results in a lower polymer-to-SPION ratio and reduced magnetic shielding [54]. Additionally, the blocking temperatures (TB) of S9 (86.16 K) and S13 (90.25 K) were lower than that of the free OLM-SPIONs (97.87 K), further confirming the successful encapsulation of the OLM-SPIONs within the polymeric matrix [55]. These findings underscore the importance of optimizing nanoparticle size and polymer composition to minimize magnetic dilution effects while ensuring sufficient magnetic responsiveness for MFH applications.

The MFH performance of SPION-encapsulated TPS-NPs (S9 and S13) demonstrated their strong potential as efficient heat-inducing agents under biologically safe AMF conditions (H×f ≈ 4.1 GAm^−1^s^−1^), remaining well within Hergt’s biological safety limit (~5 GAm^−1^s^−1^) [17]. Both samples successfully reached the therapeutic threshold of 42 °C, with S9 achieving this in 39.1 min and S13 in 49.8 min at an Fe concentration of 3 mg_Fe_/mL. The enhanced heat-generating efficiency of these TPS-NPs, as reflected in their intrinsic loss power (ILP) values (S9: 1.39 ± 0.07 nHm^2^/kg, S13: 1.31± 0.06 nHm^2^/kg), was superior to that of previously reported PVA-Stabilized PLGA Nanoparticles (PV-NPs)/TPS-NPs (Table 4). Interestingly, despite a slightly lower SPION encapsulation efficiency, S9 exhibited higher ILP and SAR values than S13, suggesting that key physicochemical properties played a crucial role in determining its superior MFH efficiency. The potential contributing factors to this observation are discussed below. The superior heating capability of S9 can be attributed to several interrelated factors. First, SQUID magnetometry analysis indicates that S9 exhibits a higher saturation magnetization (Ms) than S13, which could enhance its magnetic responsiveness under AMF exposure and lead to more efficient heat induction. This observation aligns with previous studies suggesting that higher Ms values are associated with improved MFH heating efficiency [54]. Since hyperthermia efficacy depends on nanoparticle magnetization, the superior Ms of S9 may facilitate greater energy absorption and heat conversion, reinforcing its potential as a highly efficient heat-inducing agent.

Additionally, the smaller hydrodynamic size of S9 (~144 ± 3 nm) may contribute to a higher surface-area-to-volume ratio, potentially increasing SPION exposure to the AMF. This could help minimize the magnetic shielding effect of the PLGA polymer and enhance energy transfer efficiency [54]. Moreover, the shorter heat conduction path in smaller TPS-NPs might enable faster heat dissipation, leading to more effective thermal energy transfer to the surrounding medium [56,57]. This aspect is particularly important in MFH-based cancer therapy, where localized and controlled heating is necessary to maximize tumor cell destruction while minimizing damage to surrounding healthy tissues [9]. Furthermore, the low molecular weight of PLGA in TPS-NPs may facilitate efficient thermal conduction, potentially optimizing heat transfer within the polymeric matrix [58]. The optimized size distribution and well-dispersed nature of the TPS-NPs could also contribute to efficient heat dissipation, reinforcing their suitability for MFH-based biomedical applications. Another possible contributing factor is the presence of TPGS as a stabilizer, which may help improve colloidal stability, reduce aggregation, and enhance dispersion, potentially leading to more uniform heat distribution under AMF exposure [39,58]. However, further studies are required to fully understand the extent of TPGS retention during the washing process and its impact on overall MFH performance. Although certain commercial SPION formulations, such as Resovist and Nanomag-D-spio, have demonstrated higher ILP values—up to 3.1 nHm^2^/kg—these systems are ferrofluidic in nature and stabilized by short/long-chain ligands such as dextran or starch [29]. In contrast, the SPIONs in the present study are encapsulated within PLGA, a biodegradable and FDA-approved polymer, forming a solid polymeric nanoplatform [58]. While such encapsulation may attenuate the overall magnetic saturation due to magnetic dilution effects, it offers several critical advantages, including enhanced colloidal and thermal stability, reduced aggregation, prolonged systemic circulation, and the potential for multifunctional integration such as drug co-delivery [59,60]. These properties are particularly important for clinical translation, where safety, stability, and efficacy must be balanced.

The thermogravimetric analysis (TGA) results indicate the thermal stability and composition of the SPION-encapsulated TPS-NPs, highlighting their potential suitability for biomedical applications, particularly MFH-based cancer therapy. The initial weight loss observed between 30–200 °C is likely due to the evaporation of residual moisture, a common phenomenon in polymeric nanoparticles [61,62]. The major weight loss occurring between 201–600 °C corresponds to the degradation of the PLGA matrix. The residual weight in S9 is attributed to the encapsulated Fe_3_O_4_ (OLM-SPIONs), as suggested by UV–Vis spectroscopy-based encapsulation efficiency (EE%) (refer to Table 1), supporting the successful incorporation of the OLM-SPIONs within the polymer matrix. Moreover, these findings align with previous studies on PLGA-based SPION formulations, where similar thermal degradation patterns and residual OLM-SPION content have been reported [61,63]. The observed thermal stability of the TPS-NPs further suggests that the polymer matrix may provide a protective environment for the encapsulated OLM-SPIONs, potentially minimizing premature degradation. This characteristic could be particularly beneficial for MFH-based cancer therapy, where maintaining nanoparticle stability under physiological conditions is crucial for controlled heat generation and sustained therapeutic effects.

The differential scanning calorimetry (DSC) curves of the raw PLGA (i), empty TPS-NPs (S4) (ii), and SPION-encapsulated TPS-NPs (S9) (iii) further support the thermal behavior of the system (see Figure S4). For S9, two distinct endothermic transitions were observed: a minor peak in the 38–40 °C range and a more pronounced transition near 49 °C. The first transition is attributed to the presence of residual amorphous TPGS, a surfactant retained from the emulsification process. This low-temperature event was not considered the glass transition temperature (Tg) of the system.

Instead, the second thermal transition (~49.29 °C) was selected as the Tg, as it aligns with that of raw PLGA (~46.4 °C) and S4 (~48.6 °C), confirming that it represents the main polymer matrix. The shift of Tg from 48.6 °C in S4 to 49.29 °C in S9 indicates restricted chain mobility, likely due to enhanced polymer–SPION interactions, which could contribute to the improved structural integrity and thermal stability of the nanoplatform under physiological conditions [33,34]. These characteristics are critical for MFH, where controlled heating and long-term particle stability are required.

The MFH performance of the SPION-encapsulated TPS-NPs (S9 and S13) was assessed under alternating magnetic field (AMF) conditions to evaluate their efficacy as heat-inducing agents while maintaining biocompatibility. The heating efficiency of these nanoparticles remained well within Hergt’s biological safety limit of ~ 5 GAm^−1^s^−1^, which represents the upper threshold for AMF exposure to avoid harmful eddy current heating in biological tissues. Both S9 and S13 successfully achieved the hyperthermia threshold (42 °C) necessary for effective cancer therapy, with S9 reaching this temperature in 39.1 min and S13 in 49.8 min at an Fe concentration of 3 mg_Fe_/mL. The slightly superior heating performance of S9, reflected in its higher intrinsic loss power (ILP) and specific absorption rate (SAR), underscores the role of nanoparticle size, encapsulation efficiency, and magnetization on MFH efficacy. Notably, these results were achieved using biocompatible, FDA-approved components (PLGA and TPGS) that support favorable safety profiles. To further confirm the clinical potential of our nanoplatforms, comprehensive in vitro assessments such as cell viability, reactive oxygen species (ROS) generation, Fe^3+^/Fe^2+^ release, and hemolysis assays will be conducted prior to progressing to in vivo studies. Overall, these findings highlight the potential of SPION-encapsulated TPS-NPs as a safer and more effective nanoplatform for MFH-mediated cancer therapy.

**Table 4:** Comparison of results obtained for SPION-encapsulated TPS-NPs (i.e., SPION-encapsulated TPS-NPs (S9)) with previous reported work on SPION-encapsulated polymer nanoparticles.

Sr. No	Samples	Dh (nm)	Particle Size (nm)	OLM-SPIONs /Fe (EE %)	AMF (H (kA/m) & f (KHz))	H × f (GAm^−1^ s^−1^)	Ms (emu/g)	SAR (W/g_Fe_)	ILP (nHm^2^kg^−1^)	Mini. Particles Concern. mg_Fe_/mL)	Ref.
1	SPION-encapsulated TPS-NPs	144 ± 3	140 ± 27.71	~	H = 8.6, f = 475.5	4.1	25.1	48.8 ± 2.3	1.39± 0.07	3	This work
2 ∞	CCM-NIF-SPION-encapsulated TPS-NPs	218 ± 3	194±14	73.2	H = 11.5, f = 751.5	8.6	9.1	62.8	0.6	0.5	[25]
3 a	CCM-Ver-SPION-encapsulated PV-NPs	282 ± 2.4	250-280	64.9	H = 10.9, f = 751.5	8.2	8.2	8.5	0.1	6	[27]
4	CCM-5Fu-SPION-encapsulated PV-NPs	-	150	-	H = 18.03, f = 305	5.5	-	-	-	0.5	[51]
5	CCM-SPION-encapsulated. PV-NPs	72	250	65	H = 11.7, f = 427	5	6	59	-	5	[64]
6	SPION-encapsulated. TPGS-NPs	-	178.2	-	H = 89, f = 240	21.3	73.9	51.4	-	1	[65]
7	Aminosilane-coated SPIONs	100	-		H = 16, f = 874	14	-	194	-	0.6	[66,67]
										0.1	
8	Aminosilane-coated SPIONs	-	12	-	H = 2-15, f = 100	1.5	-	-	-	112 *	[68]

Note: ∞ and a are our previously reported studies on TPS-NPs and PV-NPs. * Concentration in mg/mL.

## 5. Conclusions

This study successfully developed SPION-encapsulated TPS-NPs as efficient and biocompatible heat-inducing agents for MFH-based cancer therapy. The nanoparticles exhibited enhanced heating efficiency while remaining within Hergt’s biological safety limit (~ 5 GAm^−1^s^−1^), demonstrating their potential for clinical applications. The optimized formulation, particularly S9, showed superior encapsulation efficiency, size distribution, and magnetic properties, leading to effective heat generation under AMF exposure. These findings highlight the importance of tailored nanoparticle design for safe and efficient MFH applications, particularly in the treatment of solid tumors such as breast, prostate, or ovarian cancer. However, further in vivo studies are necessary to confirm their therapeutic efficacy, biocompatibility, and stability under physiological conditions, paving the way for future advancements in nanoplatform-based cancer therapies.

## Figures and Tables

**Table 2:** Saturation magnetization (Ms) values and blocking temperature (TB) of samples.

Sr. No.	Samples	Sample Code	Ms (emu/g)		Blocking Temperature (T_B_)
			At 10 K	At 300 K	
1	OLM-SPIONs	-	66.6 *	59.1 *	100 K
2	SPION-encapsulated TPS-NPs (0.5 Wt % TPGS)	S9	25.09 ± 0.07	21.66 ± 0.03	86.16 K
3	SPION-encapsulated TPS-NPs (1 Wt % TPGS)	S13	24.13 ± 0.06	20.64 ± 0.02	90.25 K

* Reported in previous publication.

**Table 3:** The maximum temperatures and ∆T attained within 60 min by the SPION-encapsulated TPS-NPs samples (S9 and S13) at concentrations of 1, 2, and 3 mg_Fe_/mL, and (ii) the ILP and SAR values of the SPION-encapsulated TPS-NPs samples (S9 and S13) at concentrations of 3 mg/mL.

Sr. No.	Sample Code	OLM-SPIONs Concentration (mg_Fe_)	PLGA (mg)	TPGS % (*w*/*v*)	Time (mins)	Temperature (°C)	∆T (°C)	SAR (W/g_Fe_)	ILP (nHm^2^kg^−1^)
1	S9	3 mg_Fe_	10	0.5	39.1	42.0	~12	48.8 ± 2.3 ± 2.3 ± 2.3 ± 2.3	1.39 ± 0.07
	S13		10	1	49.8	42.0		46.1 ± 2.1	1.31 ± 0.06
2	S9	2 mg_Fe_	10	0.5	60	41.5	~10	-	-
	S13		10	1	60	41.2		-	-
3	S9	1 mg_Fe_	10	0.5	60	40.7	~10	-	-
	S13		10	1	60	39.8		-	-

## Data Availability

The data presented in this study are available upon request from the corresponding author.

## Supplementary Materials

The following supporting information can be downloaded at: https://www.mdpi.com/article/10.3390/bioengineering12070715/s1.

## Abbreviations

The following abbreviations are used in this manuscript:

AFM	Atomic Force Microscopy
DLS	Dynamic Light Scattering
DSC	Differential Scanning Calorimetry
Dh	Hydrodynamic Diameter
ILP	Intrinsic Loss Power
KTP	Potassium Thiocyanate-Based Protocol
M-SESE	Modified Single Emulsion Solvent Evaporation
MFH	Magnetic Fluid Hyperthermia
OLM	Oleic Acid and Oleylamine
PLGA	Poly (D, L-lactide-co-glycolide)
PV-NPs	PVA-Stabilized PLGA Nanoparticles
SAR	Specific Absorption Rate
SPIONs	Superparamagnetic Iron Oxide Nanoparticles
OLM-SPIONs	Oleic-Acid-and-Oleylamine-coated SPIONs
SQUID	Superconducting Quantum Interference Device
TB	Blocking Temperatures
TEM	Transmission Electron Microscopy
TGA	Thermogravimetric Analysis
TPGS	D-α-Tocopheryl Polyethylene Glycol Succinate
TPS-NPs	TPGS-stabilized PLGA nanoparticles
UV–Vis	Ultraviolet–Visible
ζ	Zeta Potential
