# Supplementary material for: Unlocking Superior MFH Performance Below Hergt’s Biological Safety Limit: SPION-Based Magnetic Nanoplatforms Deliver High Heating Efficiency at Low AMF
Source: Bioengineering (Basel). 2025 Jun 30;12(7):715. doi: 10.3390/bioengineering12070715 (PMC12292120; doi:10.3390/bioengineering12070715)
Supplement: Supplementary file 1 [file bioengineering-12-00715-s001.zip › bioengineering-3623890-supplementary.pdf]

Supplementary Information

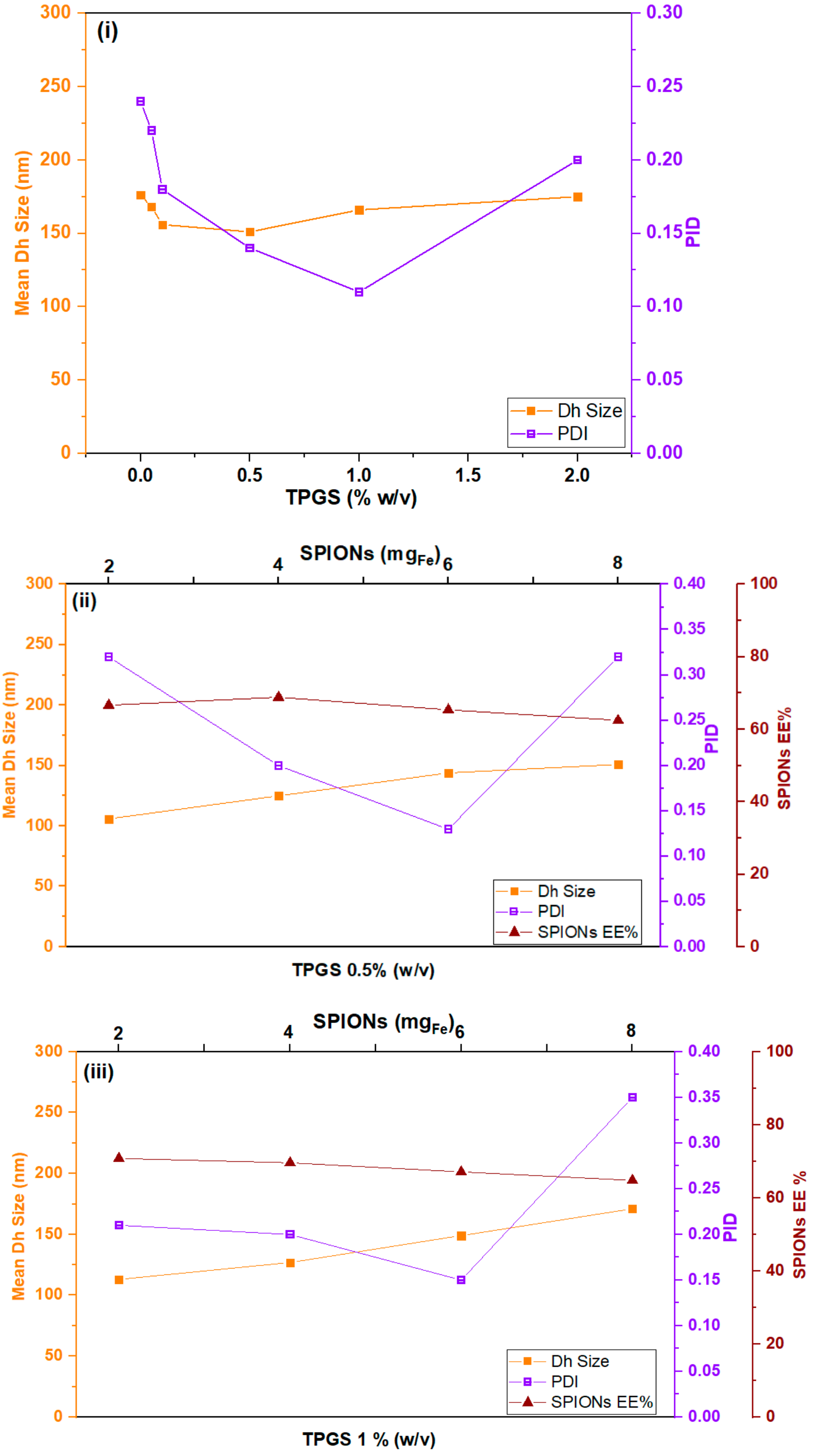

Figure S1: (i) Plot showing the variation in hydrodynamic diameter (Dh) and polydispersity index (PDI) with different concentrations of TPGS (% w/v); (ii) and (iii) plots showing the variation in Dh, PDI, and encapsulation efficiency (EE%) of SPIONs (mg_{Fe}) with increasing SPION loading in SPION-encapsulated TPS-NP formulations prepared using 0.5 wt% and 1 wt% TPGS, respectively.

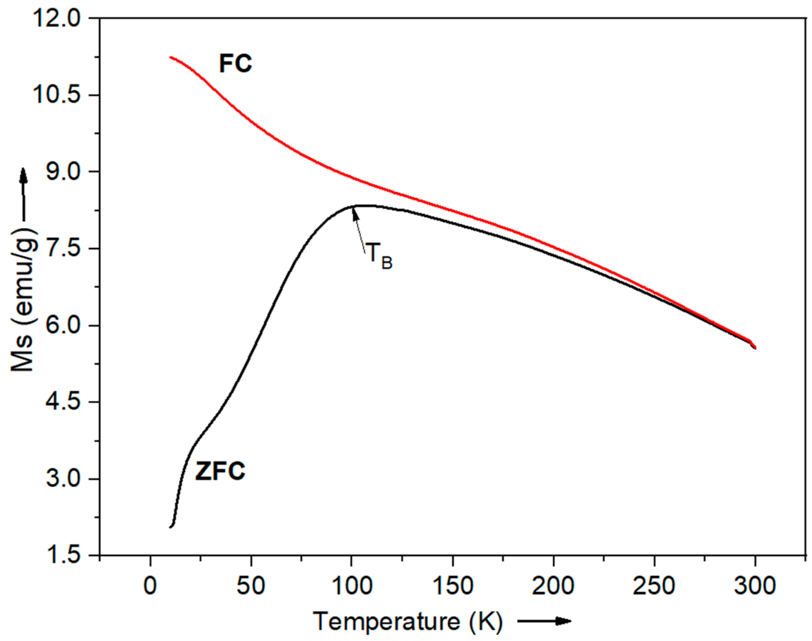

Figure S2: Field-Cooled (FC) and Zero-Field-Cooled (ZFC) magnetization curves of OLM-coated SPIONs measured under an applied magnetic field of 100 Oe. The blocking temperature (T_B) is indicated.

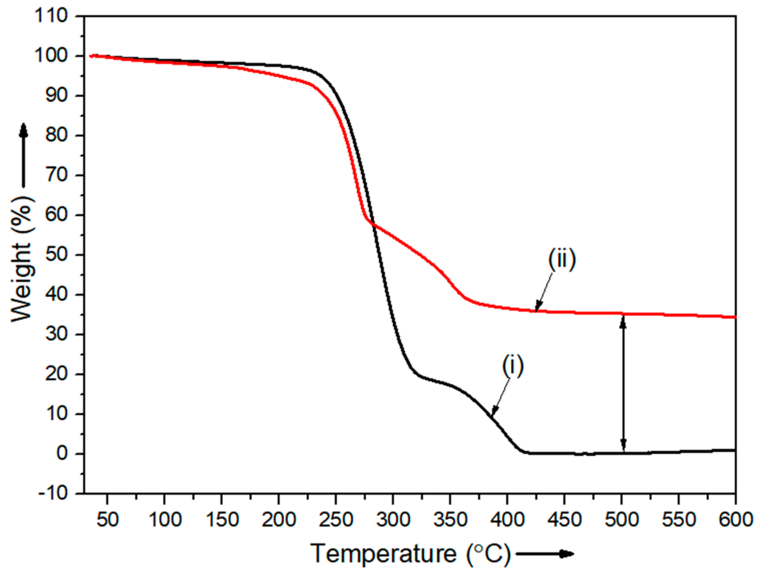

Figure S3: Displays TGA curves, of (i) empty-TPS-NPs (S4) and (ii) SPIONs-encapsulated TPS-NPs (S9) synthesized via 0.5% Wt TPGS concentration.

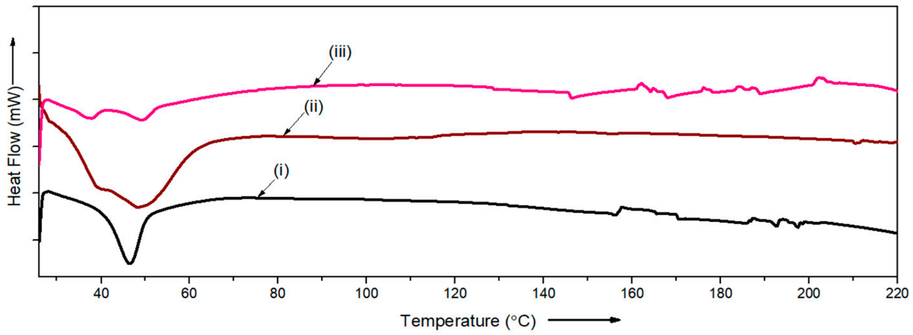

Figure S4. Illustrates DSC graphs for different samples: (i) raw PLGA polymer, (ii) empty-TPS-NPs (S4), and (iii) SPIONs-encapsulated TPS-NPs (S9).
